# Association between ambient temperature, particulate air pollution and emergency room visits for conjunctivitis

**DOI:** 10.1186/s12886-021-01854-1

**Published:** 2021-02-24

**Authors:** S. Khalaila, T. Coreanu, A. Vodonos, I. Kloog, A. Shtein, L. E. Colwell, V. Novack, E. Tsumi

**Affiliations:** 1 Department of Ophthalmology, Soroka University Medical Center, P.O. Box 151, 84101 Beer-Sheva, Israel; 2 Faculty of Health Sciences, Ben-Gurion University of the Negev, Beer-Sheva, Israel; 3 Negev Environmental Health Research Institute, Soroka University Medical Center, P.O. Box 151, 84101 Beer-Sheva, Israel; 4 Department of Geography and Environmental Development, Ben-Gurion University of the Negev, Beer-Sheva, Israel; 5 University of Massachusetts Medical School, Worcester, MA USA

**Keywords:** Conjunctivitis, Temperature, Weather, Air pollution, Ocular disease

## Abstract

**Background:**

Numerous studies have confirmed the association of ambient temperature and air pollution with a higher risk of morbidities, yet few have addressed their effect on the ocular system. The purpose of this study was to assess the association between temperature, air pollution, and emergency room visits for conjunctivitis.

**Methods:**

In this case-crossover study, the records of all emergency room visits to Soroka University Medical Center (SUMC) from 2009 to 2014 were reviewed for patients with conjunctivitis. Daily exposure to fine and coarse particulate matter and temperature were determined by a hybrid model involving satellite sensors. Mean relative humidity was obtained from the Ministry of Environmental Protection meteorological monitoring station located in Beer-Sheva.

**Results:**

Six hundred one patients were diagnosed with conjunctivitis in the SUMC emergency room. We discovered a positive association between temperature increments and incidence of conjunctivitis. The strongest effect was found during summer and autumn, with an immediate (lag0) incidence increase of 8.1% for each 1 °C increase in temperature (OR = 1.088, 95%CI: 1.046–1.132) between 24 and 28 °C in the summer and 7.2% for each 1 °C increase in temperature (OR = 1.072, 95%CI: 1.036–1.108) between 13 and 23 °C in the autumn. There was no statistically significant association between fine and coarse particulate matter and conjunctivitis incidence.

**Conclusion:**

Temperature increases during summer and autumn are significantly associated with an increased risk of conjunctivitis. Conjunctivitis is not associated with non-anthropogenic air pollution. These findings may help community clinics and hospital emergency rooms better predict conjunctivitis cases and will hopefully lead to improved prevention efforts that will lower the financial burden on both the individual and the public.

**Supplementary Information:**

The online version contains supplementary material available at 10.1186/s12886-021-01854-1.

## Background

Conjunctivitis is an inflammation of the conjunctiva characterized by swelling, redness, discharge, and discomfort. It is a common diagnosis in the general population [7] and the most common ocular condition diagnosed in United States’ emergency rooms where it accounts for almost one-third of all eye-related visits [10]. Conjunctivitis scan be infectious (generally caused by adenovirus) or noninfectious (autoimmune, hypersensitivity, etc.).

Conjunctivitis significantly impacts health-care systems; outbreaks can cause significant morbidity, high health-care costs, and loss of workdays, which result in financial burdens on both individuals and the public worldwide [7, 10, 36]. In the United States, 4–6 million conjunctivitis visits annually entail nearly 800 million dollars in treatment costs [36]. The direct effects of the condition on patients’ quality of life can vary, ranging from lost school/work to irreversible eye and vision damage. More information about environmental risk factors is crucial to develop measures to reduce the incidence and burden on public health of conjunctivitis [11].

Ambient temperature and air pollution are known to be associated with a variety of health problems and disorders that affect multiple body systems, such as respiratory, cardiovascular, and neurological systems [1, 2, 4, 15, 17, 28, 41–45]. In severe cases, temperature and air pollution can be fatal to individuals [1, 20, 21, 25]. Several studies have revealed meteorological effects on the ocular system [6, 9, 14, 19, 30, 37, 39], but few agree about the relationship of meteorological changes with conjunctivitis [5, 12, 13, 18, 40].

Chia-Jen et al. [12] reported more outpatient visits for conjunctivitis in winter than in summer; however, Chiang et al. [13] reported that average daily visits for acute conjunctivitis peaked in the summer. According to Szyszkowicz et al. [40], the number of visits was higher in the warm season than the cold season. Azari and Barney [7] reported a greater incidence of viral conjunctivitis in summer and bacterial conjunctivitis from December through April. These studies all evaluated the incidence of conjunctivitis by season rather than by temperature.

The present study was undertaken to evaluate the association between air pollution, ambient temperature, and emergency room visits for conjunctivitis in the Negev Desert of southern Israel. This 13,000 km^2^ semi-arid region lies between the Saharan and Arabian deserts and the three together constitute the world’s largest dust belt. In the light of climate change and desertification, the Negev can be considered a predictor of future climate change in many regions of Europe.

## Methods

### Study population

The study enrolled all patients who arrived at the Soroka University Medical Center (SUMC) Emergency room during the years 2009–2014 and were diagnosed with conjunctivitis. Patients with other eye disorders were used to determine incidence of conjunctivitis compared with other eye complaints. SUMC is a tertiary 1000-bed hospital in the Negev Desert, and the only medical center in the region for a population of 700,000 inhabitants. The Center is owned by the largest health maintenance organization HMO in Israel, Clalit Health Services. Only residents of the southern Negev were enrolled. Demographic and clinical data obtained from the electronic database of Clalit Health Services included date of birth, gender, age, place of residence, eye diseases, and comorbidities.

### Air pollution and meteorology data measurement

Seasons were defined according to Alpert et al. [3]: winter (December 7–March 30), summer (May 31–September 22)—each lasts about 4 months—autumn (September 23–December 6), and spring (March 31–May 30)—each lasting approximately 2 months.

#### Air pollution

There are two main source of air pollution: naturally occurring non-anthropogenic pollutants (such as volcanic eruptions, forest fires, and dust) and anthropogenic air pollutants created by human activity (such as O_3_, NO_2_, NOX, and SO_2_) [34].

We used a hybrid method for assessing spatiotemporal exposure to PM_10_ (particulate matter 10 μm or less in diameter) and PM_2.5_ (particulate matter 2.5 μm or less in diameter) [22, 23, 33, 38]. Daily average concentrations were estimated using a hybrid satellite-based model that provides daily satellite remote sensing data at 1 × 1 km spatial resolution [23, 38] according to the residential address of each patient. Briefly, using the multi-angle implementation to atmospheric correction (MAIAC) algorithm [27], which was developed by NASA and provides aerosol optic depth (AOD) data in high resolution, we applied mixed models to regress daily PM_10_ and PM_2.5_: AOD, traditional land use regression, and temporal and spatial predictors. When AOD was not available, we fitted a generalized additive model with a thin plate spline term of latitude and longitude to interpolate PM estimates. Good model performance was achieved, with out-of-sample cross validation R^2^ values of 0.92 and 0.87 for PM_10_ and PM_2.5_, respectively. Model predictions had little bias, with cross-validated slopes (predicted vs. observed) of close to 1 for both models. Exposure estimates were assigned for each patient based on his/her geocoded home address. The estimates provided for PM10 are per 10 μg/m3 changes in air pollution.

#### Meteorological data

Daily data on mean air temperature were obtained by a satellite-based model, recently installed by the Israel Meteorological Service for monitoring temperature in the Negev, that provides daily satellite remote sensing data at 1 × 1 km spatial resolution for each patient. Mean relative humidity was obtained from the Ministry of Environmental Protection Meteorological monitoring station located in Beer-Sheva, the location of SUMC and the largest city in southern Israel.

## Statistical analysis

The association between air pollution, ambient temperature, and emergency room visits for conjunctivitis was examined using a case-crossover design [29, 31, 32, 44, 46]. This design samples only cases and compares each case’s exposure to temperature and particulate air pollution during a time period just before the case-defining event (hazard period) with the subject’s own exposure in other reference periods (control periods). The hazard period was chosen to be 6 days as this matches the incubation period of conjunctivitis, which in most cases is up to 5–6 days. Because each subject serves as his or her own control, there is perfect matching on all measured or unmeasured subject characteristics that do not vary over time.

We used a symmetric bidirectional case-crossover design. In this design, reference periods are symmetrically spaced in time, both before and after the hazard period, which minimizes potential confounding by season or time trends. In this study, the hazard period was defined as the day of admission; temperature and particulate air pollution exposure was modeled as the exposure on the day of admission and a mean exposure for the 7 days prior to admission. Matched strata were constructed for each subject (that is, admission day) consisting of the event day (day of admission) and six matched control days. These days were chosen to be days 7, 14, 21, and 28 before the event and days 7, 14, 21, and 28 after the event.

The statistical analysis included two steps. First, conditional logistic regression [8] models were used to test a possible non-linear association of daily mean temperature with incidence of conjunctivitis for lag 0 to lag 6 and the cumulative effect of 7 days. To allow a non-linear effect of temperature we used penalized splines with five degrees of freedom for temperature and PM_10_. Models were adjusted for relative humidity as a linear term.

Next we performed a conditional logistic regression to calculate odds ratios (OR) and 95% confidence intervals (CI) for lag 0 to lag 6 on risk of conjunctivitis with the main exposure daily mean temperature controlled for PM_10_ and humidity. Stratified analysis by season (fall, winter, spring, summer), gender (female, male), and age (0–18 years old, 18–65 years old, and over 65 years old) was performed.

Statistical analysis was performed with the IBM SPSS Statistics software package (version 23.0, IBM, Armonk, NY) and R statistical software (version 3.5.1).

The study was conducted according to the Declaration of Helsinki and approved by the Medical Helsinki Committee of the Soroka University Medical Center, Ben-Gurion University of the Negev.

## Results

### Population

Of 19,264 visits to the ophthalmology SUMC emergency room between 2009 and 2014, 6001 (31.1%) were diagnosed with conjunctivitis (Table 1 in the appendix). The mean age of the patients was 34.6 years (range 0.5–96 years) and 54.4% were male (Table 2 in the appendix). The majority of patients, 4063 (67.7%), were between 16 and 65 years of age.

**Table 1:** Air pollution and meteorology values between the years 2009 and 2014

		Winter^a^Dec 7–Mar 30	Spring^a^Mar 31–May 30	Summer^a^May 31–Sep 22	Autumn^a^Sep 23–Dec 6
**PM**_**2.5**_ μg/m^3^	IQR	17.52–24.82	17.38–23.14	18.11–22.05	17.21–22.91
	Mean ± SD	22.62 ± 8.30	21.34 ± 6.42	20.46 ± 3.74	20.60 ± 5.21
	Minimum	5.28	8.33	9.69	5.28
	Maximum	53	53	53	53
**PM**_**10**_ μg/m^3^	IQR	39.10–56.66	40.13–51.62	40.39–47.04	38.44–52.68
	Mean ± SD	56.18 ± 34.35	49.51 ± 20.94	44.40 ± 8.92	47.96 ± 17.95
	Minimum	11.53	21.08	40.39	11.53
	Maximum	193	193	193	193
**Temperature** ^o^C	IQR	11.86–15.65	18.64–20.95	25.06–27.32	17.02–23.39
	Mean ± SD	13.94 ± 3.42	20.90 ± 3.33	26.14 ± 1.81	20.16 ± 4.40
	Minimum	3.55	7.34	16.55	4.15
	Maximum	28.11	30.87	33.62	29.80
**Relative humidity (%)**	IQR	57.00–75.00	52.00–71.50	63.00–71.54	57.00–71.40
	Mean ± SD	65.43 ± 13.83	59.66 ± 13.68	66.46 ± 8.20	62.70 ± 13.40
	Minimum	15.53	13.00	17.00	13.00
	Maximum	92.45	81.77	81.77	93.14

^a^ Seasons defined according to Alpert et al. [3]

### Pollution and meteorology

The average daily levels of meteorological and air pollution variables by seasons are presented in Table 1. The interquartile range (IQR) of PM_2.5_ was 17.21–24.82 μg/m^3^ and the maximum value reached was 53 μg/m^3^, with no significant differences between seasons. The IQR of PM_10_ was 38.44–56.66 μg/m^3^ and the maximum value reached was 193 μg/m^3^; it tended to be slightly higher during the winter. The climate in the study region is relatively hot and dry, with Köppen climate classification type Csa [35]. Average daily IQR temperatures are 11.86–15.65 °C in winter, 18.64–20.95 °C in spring, 25.06–27.32 °C in summer, and 17.02–23.39 °C in autumn. The IQR relative humidity range was 57.00–75.00%, with no difference between seasons.

### Effect of air pollution on the incidence of conjunctivitis

ORs and 95% CIs were estimated from conditional logistic regression analysis of association between PM_2.5_, PM_10_ (separately), temperature, and emergency room visits due to conjunctivitis: models were adjusted for humidity. There was no statistically significant correlation between levels of PM_2.5_ or PM_10_ and the incidence of conjunctivitis (Table 3 in the appendix).

### Effect of temperature on the incidence of conjunctivitis

**Fig. 1:**
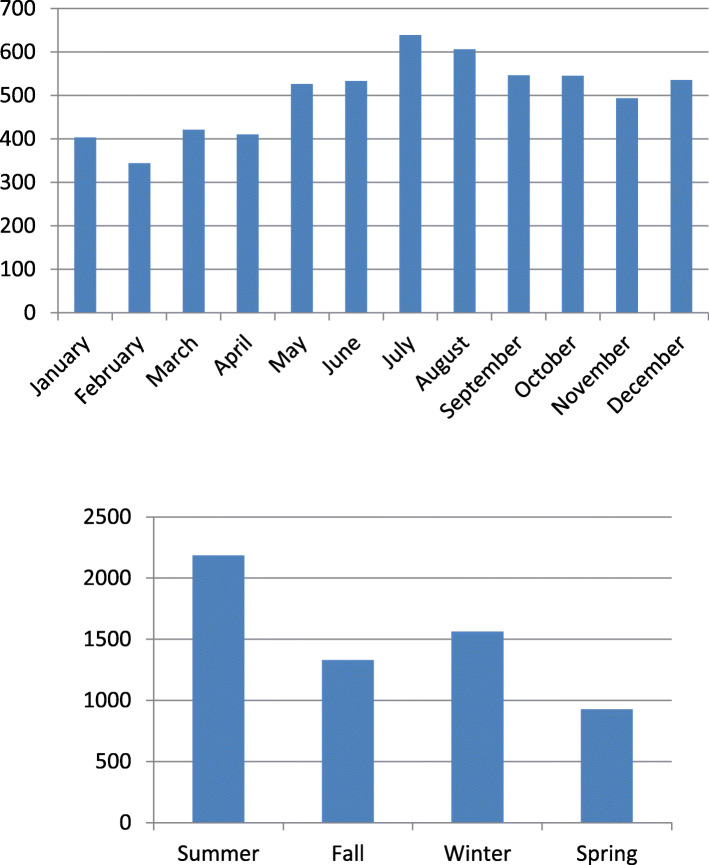
Rate of conjunctivitis by **a**. season and **b**. month

A subgroup analysis was conducted to assess the seasonal relationship between temperature and conjunctivitis. The number of emergency room visits for conjunctivitis was higher in summer (36.4%, *p* < 0.001) than in winter (26.0%, p < 0.001), autumn (22.1%, *p* = 0.773), and spring (15.4%, *p* = 0.135), with the highest rates recorded in July and August (Fig. 1) (Table 1 in the appendix). When the incidence of conjunctivitis was compared with other ophthalmological disorders by season (Table 1 in the appendix), it was found to be significantly higher in summer (36.4 and 32.8%, respectively, *p* < 0.001) and significantly lower in winter (26.0 and 28.6%, respectively, p < 0.001).

The same results were obtained when the data were stratified by age, with summer leading in each age group (p < 0.001). When analyzed by month, July and August had the highest incidence for each age group (Fig. 1 and Table 4 in the appendix). Multivariate analysis showed no association between the incidence of conjunctivitis and age.

Women were found to have a significant association between temperature and conjunctivitis on all seven day lags and men were found to have a significant association on lags 0 and 6 (Table 5 in the appendix).

**Fig. 2:**
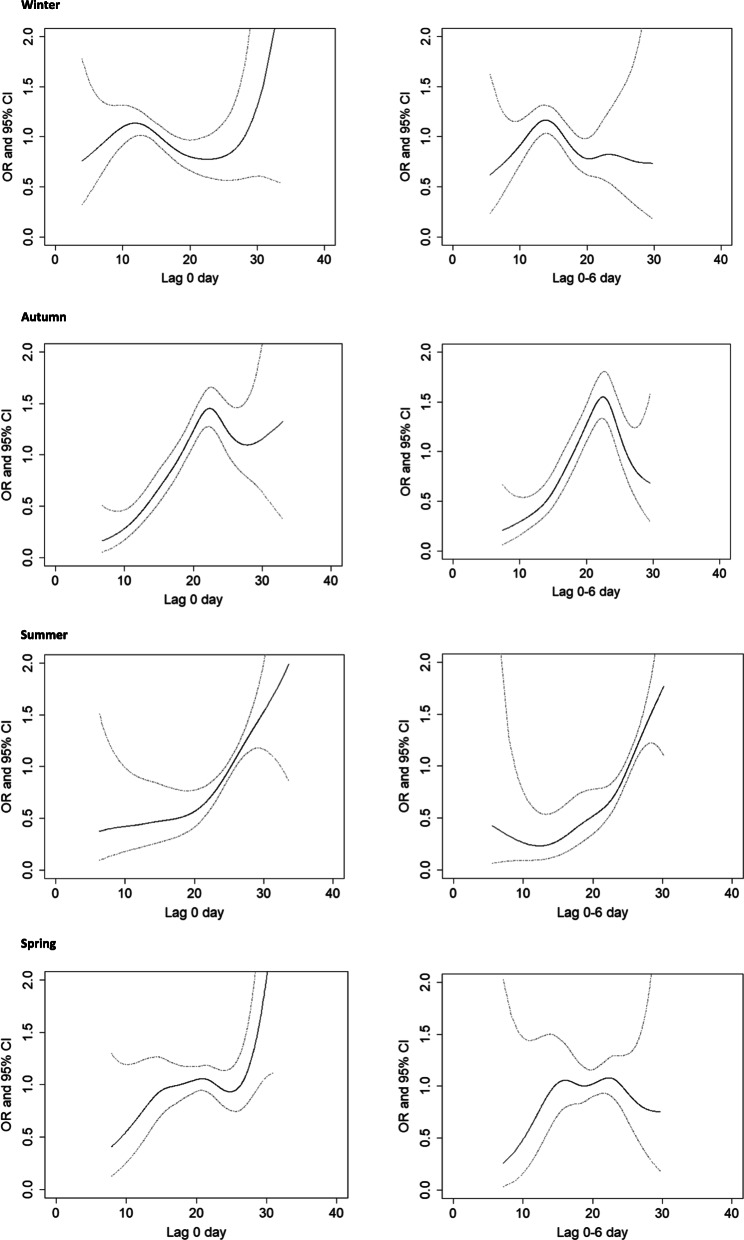
Association between increase in temperature and visits to the emergency room for conjunctivitis, by season. The solid lines are the odds ratios (ORs) and the dotted lines are 95% confidence intervals. Models were adjusted for humidity and PM_10_

There is an overall non-linear association between temperature and conjunctivitis. However, for certain temperature ranges in summer and autumn, we noticed a form of linear positive association between temperature increment and incidence of conjunctivitis (Fig. 2). In summer, the incidence of conjunctivitis increased by 8.1% (95%CI: 1.046–1.132) with each 1 °C rise in temperatures between 24 and 28 °C, and for autumn the incidence increased by 7.2% (95%CI: 1.036–1.108) with each 1 °C rise in temperatures between 13 and 23 °C. This association remained after taking into account a 6-day lag between exposure and developing conjunctivitis for both autumn and summer. There was no association between temperature and conjunctivitis during spring and winter (Fig. 2).

## Discussion

The main result of our study is the significant association between temperature and the incidence of conjunctivitis during a range of temperatures in summer and autumn. These temperature ranges (13–23 °C in autumn and 24–28 °C in summer) are considered the normal temperatures for most days in these specific seasons, with very few days having temperatures outside of these ranges. Thus, there were no reliable statistical conclusions outside these ranges. No association was found between air pollution (PM_2.5_, PM_10_) and the incidence of conjunctivitis.

Meteorological changes and air pollution have been a public health concern for several decades due to accelerated global warming and desertification. Contemporary studies document the effect of temperature and air pollution on human morbidities, especially cardiovascular, respiratory, and neurological systems, as well as on human mortality, even within the normal range of temperatures [1].

Several studies have investigated the association between meteorological changes and ocular diseases. Hu et al. [19] found that primary angle closure glaucoma admission rates became significantly higher with increased relative humidity but showed no correlation to temperature. Matthew et al. [30] reported a higher frequency of infectious keratitis during the higher temperatures and humidity levels of summer. Even an increased risk of tractional retinal detachment was linked to elevated outdoor temperatures by Augera et al. [6]. Furthermore Christoph et al. [14] supported a correlation between higher weekly average temperature and increased ophthalmology emergency room visits.

Few studies have investigated the effect of temperature on conjunctivitis: even those studies examining seasonal conjunctivitis incidence have not accounted for temperature directly [13, 18, 40].

The pathophysiological mechanisms of air pollution and meteorological changes on conjunctiva remain to be characterized. Some studies [16, 26] have speculated that PM_2.5_ and PM_10_ particles cause intraocular epidermal cells to lose their ability to adapt, leading to cell death and inflammation. Krishna et al. [24] pointed to the strong oxidative stress effect of NO_2_ and O_3_ that may stimulate conjunctival cell inflammation. Although various authors have related subtypes of conjunctivitis to specific seasons—viral conjunctivitis is common in summer, bacterial in winter, allergic in spring—there is no consensus among them on the exact mechanisms in play [7, 13, 18, 40].

In the Negev Desert, there is little anthropogenic (chemical) pollution, supporting our finding that non-anthropogenic air pollution is not related to conjunctivitis without the confounding effect of anthropogenic pollution. However, other studies in other locations may have had chemical pollution confound the effect of PM on the incidence of conjunctivitis.

Our findings of seasonal differences in the incidence of conjunctivitis agree with those of Hong and co-workers [18]: higher levels of temperature and lower humidity lead to increased outpatient visits for allergic conjunctivitis, which is potentially due to pollen production in warmer temperatures. In our study, we investigated the meteorological effect on non-specific conjunctivitis whereas the Hong et al. study targeted allergic conjunctivitis. Furthermore, our study measured exact temperatures in relation to each patient’s condition whereas Hong et al. grouped patients by season. Chiang et al. [13] found the incidence of chronic conjunctivitis to peak in summer, more so in rural than urban areas, a difference they attributed to factors such as socioeconomic status, income, and occupation. Szyszkowicz et al. [40] found that the number of visits to the emergency room due to conjunctivitis was higher in warm seasons (58%) than cold seasons (42%), which was also seen in our study. However, these authors studied the effect by season and not, as we did, by temperature.

The statistically significantly higher incidence of conjunctivitis in summer compared with winter suggests that higher temperatures are a risk for conjunctivitis and lower temperatures are protective against conjunctivitis. We determined that certain temperature ranges are associated with the incidence of conjunctivitis in summer and autumn. However, the lack of association between temperature and conjunctivitis in spring (moderate climate), and winter (cold climate) indicates that there is a multifactorial relationship and other factors involved. Further research is needed to understand more of these factors.

## Limitations

Although our study focuses on a specific climatic region with dry, hot, semi-desert conditions and involves a limited population, ours is a tertiary hospital and the only one in the region. We did not select the sample for the study but included the entire population.

## Conclusions

Temperature is significantly associated with conjunctivitis in southern Israel during summer and autumn. The incidence of conjunctivitis is significantly higher than other eye disorders in summer and lower in winter. There is no association between non-anthropogenic air pollution and conjunctivitis.

These findings can help community clinics and hospital emergency rooms prepare for the upticks in conjunctivitis following acute rises in temperature during certain seasons.

## Supplementary Information

**Additional file 1.**

## Data Availability

The raw data that support the findings of this study are available from Soroka Medical Database Center but restrictions apply to the availability of these data, which were used under license for the current study, and so are not publicly available. Data are however available from the authors upon reasonable request and with permission of Soroka Medical Database Center. All other data generated or analyzed during this study are included in this article and its supplementary information files.

## Abbreviations

AOD: Aerosol optic depth
PM: Particulate matter
IQR: Interquartile range
O_3_: Trioxygen
NO_2_: Nitrogen dioxide
NOX: Nitric oxide
SO_2_: Sulfur-dioxide
