# Supplementary material for: Association between ambient temperature, particulate air pollution and emergency room visits for conjunctivitis
Source: BMC Ophthalmol. 2021 Feb 24;21:100. doi: 10.1186/s12886-021-01854-1 (PMC7903634; doi:10.1186/s12886-021-01854-1)
Supplement: Supplementary file 1 — Additional file 1. [file 12886_2021_1854_MOESM1_ESM.docx]

**Appendix**

Table 1: All visits to the ophthalmology emergency room of the Soroka University Medical Center between the years 2009 and 2014

| Season | Conjunctivitis N=6,001 | Other ophthalmological disorder*  N=13,263 | p value |
| --- | --- | --- | --- |
| Summer % (n) | 36.4% (2,184) | 32.8% (4,345) | <0.001 |
| Autumn % (n) | 22.1% (1,329) | 22.3% (2,962) | 0.773 |
| Winter % (n) | 26% (1,561) | 28.6% (3,794) | <0.001 |
| Spring % (n) | 15.4% (927) | 16.3% (2,162) | 0.135 |

*All visits to the ophthalmology emergency room for reasons other than conjunctivitis

Table 2: Demographic characteristics of the 6001 patients with conjunctivitis.

| **Demographic characteristic** | **Conjunctivitis N=6001** |
| --- | --- |
| Age (mean±SD), years | 34.62±21.8 |
| 0–18 years n (%) | 1,346 (22.4) |
| 19–65 years, n (%) | 4,064 (67.7) |
| ≥66 years, n (%) | 590 (9.8) |
| Male, n (%) | 3,263 (54.4) |

# Table 3a: Odds ratios and 95% confidence intervals for emergency room visits for conjunctivitis associated with increase in 10 units of PM_10_, and mean temperature

|  |  | OR | 95% CI | p value |
| --- | --- | --- | --- | --- |
| LAG 0 | PM_2.5_ | 0.995 | 0.939; 1.055 | 0.880 |
|  | Temperature | 1.028 | 1.011; 1.045 | <0.001 |
| LAG 1 | PM_2.5_ | 0.971 | 0.914; 1.032 | 0.346 |
|  | Temperature | 1.023 | 1.006; 1.041 | 0.006 |
| LAG 2 | PM_2.5_ | 0.970 | 0.913; 1.031 | 0.327 |
|  | Temperature | 1.019 | 1.002; 1.037 | 0.025 |
| LAG 3 | PM_2.5_ | 0.971 | 0.914; 1.033 | 0.361 |
|  | Temperature | 1.019 | 1.003; 1.036 | 0.019 |
| LAG 4 | PM_2.5_ | 1.016 | 0.956; 1.080 | 0.599 |
|  | Temperature | 1.014 | 0.997; 1.030 | 0.096 |
| LAG 5 | PM_2.5_ | 0.984 | 0.925; 1.047 | 0.619 |
|  | Temperature | 1.023 | 1.007; 1.040 | 0.004 |
| LAG 6 | PM_2.5_ | 0.922 | 0.866; 0.982 | 0.011 |
|  | Temperature | 1.029 | 1.013; 1.046 | <0.001 |

# Table 3b: Odds ratios and 95% confidence intervals for emergency room visits for conjunctivitis associated with increase in 10 units of PM_10_, and mean temperature

| LAG 0 | PM_10_ | 0.985 | 0.970; 1.001 | 0.112 |
| --- | --- | --- | --- | --- |
|  | Temperature | 1.032 | 1.015; 1.049 | <0.001 |
| LAG 1 | PM_10_ | 0.987 | 0.971; 1.003 | 0.112 |
|  | Temperature | 1.025 | 1.008; 1.042 | 0.003 |
| LAG 2 | PM_10_ | 0.994 | 0.979; 1.009 | 0.446 |
|  | Temperature | 1.018 | 1.003; 1.035 | 0.017 |
| LAG 3 | PM_10_ | 0.989 | 0.973; 1.006 | 0.221 |
|  | Temperature | 1.020 | 1.003; 1.037 | 0.015 |
| LAG 4 | PM_10_ | 1.007 | 0.990; 1.023 | 0.423 |
|  | Temperature | 1.013 | 0.997; 1.030 | 0.109 |
| LAG 5 | PM_10_ | 1.004 | 0.988; 1.020 | 0.612 |
|  | Temperature | 1.021 | 1.004; 1.038 | 0.010 |
| LAG 6 | PM_10_ | 0.976 | 0.959; 0.992 | 0.004 |
|  | Temperature | 1.031 | 1.013; 1.047 | <0.001 |

Table 4: Emergency room visits for conjunctivitis according to gender, season, and age

| p value | ≥66 years  (n=590) | 19–65 years  (n=4,064) | 0–18 years  (n=1,346) |  |
| --- | --- | --- | --- | --- |
| <0.001 | 44.2 (261) | 54.1 (2,200) | 59.5 (801) | Male, % (n) |
| 0.004 | 35.4 (209) | 35.3 (1,433) | 40.3 (542) | Summer, % (n) |
| 0.501 | 20.7 (122) | 22.5 (916) | 21.5 (290) | Autumn, % (n) |
| 0.102 | 26.9 (159) | 26.6 (1,082) | 23.8 (320) | Winter, % (n) |
| 0.338 | 16.9 (100) | 15.6 (633) | 14.4 (194) | Spring, % (n) |

# Table 5: Odds ratios and 95% confidence intervals for ED visits due to conjunctivitis associated with temperature, by gender

| **Mean Temperature** | **Male** | | | **Female** | | |
| --- | --- | --- | --- | --- | --- | --- |
|  | **OR** | **95% CI** | **p value** | **OR** | **95% CI** | **p value** |
| **LAG 0** | 1.029 | 1.007;1.053 | 0.010 | 1.035 | 1.010;1.061 | <0.001 |
| **LAG 1** | 1.010 | 0.997;1.004 | 0.078 | 1.030 | 1.005;1.056 | 0.016 |
| **LAG 2** | 1.013 | 0.990;1.036 | 0.259 | 1.025 | 1.001;1.051 | 0.040 |
| **LAG 3** | 1.010 | 0.987;1.033 | 0.382 | 1.031 | 1.006;1.056 | 0.011 |
| **LAG 4** | 1.003 | 0.981;1.025 | 0.760 | 1.026 | 1.001;1.051 | 0.038 |
| **LAG 5** | 1.018 | 1.995;1.040 | 0.114 | 1.025 | 1.001;1.051 | 0.040 |
| **LAG 6** | 1.031 | 1.008;1.054 | <0.001 | 1.030 | 1.006;1.056 | 0.013 |

*Odds ratios and 95% confidence intervals were estimated from conditional logistic regression analysis of association between temperature and ED visits due to conjunctivitis. Models were adjusted for humidity and PM_10_.

Figure 1: A. Rate of conjunctivitis by season according to age. B. Rate of conjunctivitis by month according to age
